# SlicerTMS: Interactive Real-time Visualization of Transcranial Magnetic Stimulation using Augmented Reality and Deep Learning

**Published:** 2023-05-23

**Authors:** Loraine Franke, Tae Young Park, Jie Luo, Yogesh Rathi, Steve Pieper, Lipeng Ning, Daniel Haehn

**Affiliations:** 1 University of Massachusetts Boston, Boston, USA; 2 Harvard Medical School, Boston, USA; 3 Korea University of Science and Technology, Seoul, Republic of Korea; 4 Isomics, Inc., Cambridge, USA

**Keywords:** Neuronavigation, Transcranial Magnetic Stimulation, Visualization, AI, Electric Field, Virtual Reality

## Abstract

Transcranial magnetic stimulation (TMS) is a non-invasive neuromodulation approach that effectively treats various brain disorders. One of the critical factors in the success of TMS treatment is accurate coil placement, which can be challenging, especially when targeting specific brain areas for individual patients. Calculating the optimal coil placement and the resulting electric field on the brain surface can be expensive and time-consuming. We introduce SlicerTMS, a simulation method that allows the real-time visualization of the TMS electromagnetic field within the medical imaging platform 3D Slicer. Our software leverages a 3D deep neural network, supports cloud-based inference, and includes augmented reality visualization using WebXR. We evaluate the performance of SlicerTMS with multiple hardware configurations and compare it against the existing TMS visualization application SimNIBS. All our code, data, and experiments are openly available: https://github.com/lorifranke/SlicerTMS

## 1 Introduction

Transcranial magnetic stimulation (*TMS*) [4] is a powerful non-invasive brain stimulation technique (*NIBS*). TMS is used to treat various types of disorders such as major depressive disorders, migraines, clinical research on Parkinson’s or Alzheimer’s disease [13,6,2]. TMS works by placing a field generator, a *TMS coil*, close to a patient’s scalp to induce a current pulse. The pulse produces a magnetic and electric field (*E-field*) through electromagnetic induction. The E-field stimulates particular brain regions by exciting or inhibiting neurons trans-synaptically. This technique can also produce brain mappings to investigate brain circuits and their physiological properties [10,15]. However, TMS requires accurate and customized targeting of patient-specific brain connectivity to improve individualized TMS treatment efficacy [3,5]. Before the treatment, MRI data of an individual subject is required to illustrate the position between the TMS coil and the brain. Estimating the E-field at this coil position provides valuable information for identifying the stimulation target to enhance brain mapping accuracy for optimal coil placement. Therefore, deep learning techniques have recently been used to accelerate this E-field prediction. Although several techniques were developed, such as the finite element method and the boundary element method [21,9,27], most require complex head models or long computation time, making them not suitable in scenarios when real-time E-field prediction and immediate visualization are required to optimize treatment response. However, most deep learning methods are developed by directly using high-performance graphics processing unit (*GPU*) workstations, usually unavailable in a TMS clinic. Currently, no open-source software integrates deep learning-based E-fields with a neuronavigation system to make real-time E-field prediction visually accessible in a clinical environment. Since non-invasive brain stimulation has gained popularity, the development of precise neuronavigation software plays a critical role. Neuronavigation visualization systems usually include a camera-based position-tracking system and an image-data visualization platform. Recently, [14] and [16] tested neuromodulation in real-time for ultrasound, including optical tracking using the open-source platform 3D Slicer, a popular open-source medical imaging platform with a large community [7]. Current TMS visualization tools only allow manual coil placement [20] while others statically visualize the magnetic or electric field of the coil on the brain surface [1,18] with long calculation and processing times.

This paper introduces SlicerTMS, the first open-source software for real-time E-field prediction and visualization evaluated on multiple platforms. An essential contribution of this work is a framework that allows remote GPU servers to stream deep-learning E-field images to a TMS clinic where a local neuronavigation system tracks changes in the coil positions in real-time. Additionally, our work contributes through the integration of Virtual and Augmented Reality (*VR/AR* or *XR*) technologies. In the last decade, VR and AR technologies have increased in education, training, and surgical guidance in medical applications. TMS is another application that could benefit from the potential of AR’s augmented feedback, e.g., enabling users to view and interact directly with MRI images and TMS coil for increased focality and penetration of the E-Field. Only scarce open-source research is currently available to visualize TMS with virtual reality techniques [22], mainly because only recently XR is more accessible, affordable, and robust on different types of devices. Some approaches explore VR headsets [11,19,17] for coil placement or tracking, but mostly with a trade-off between coil placement accuracy and the neuronavigation software. We explored the utility of a smartphone-based AR user interface to implement a tracking component for the coil and to view the brain surface. Finally, we examined the performance of our system in an actual TMS clinic with integrated optical tracker systems to simulate a real-world use case scenario (Supplementary Material), and we evaluated SlicerTMS’ performance and functionality as a real-time system using a variety of hardware devices with data of ten different subjects.

## 2 Implementation of SlicerTMS

**Fig. 1.**
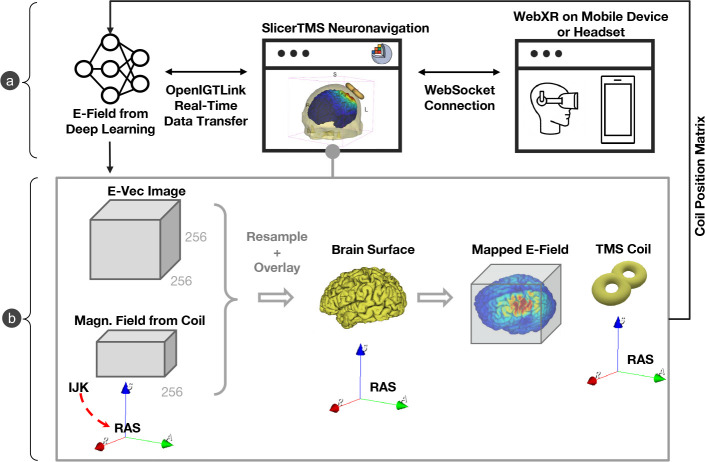
**a) Components of SlicerTMS**: Convolutional Neural Network (left) predicts E-field and transfers fields to SlicerTMS via OpenIGTLinkIF to visualize it in real-time. The WebSocket supports browser connection to WebXR to interact with the visualization in Augmented Reality (right). **b) Neuronavigation Visualization Process:** The incoming magnetic vector field image is transformed according to the TMS coil position, which is then overlayed with the brain mesh. The critical point was to consistently rotate the vector direction in each voxel as the rigid transform. The 3D TMS coil object can be moved interactively while sending the new coil positioning matrix back to the neural network to generate a new E-field.

Our TMS simulation framework consists of three components in a client-server architecture (Figure 1). The server runs the artificial neural networks for E-field prediction and the client module, which is the primary user interface of SlicerTMS. In addition, a WebXR module integrates AR devices for further visualization and user interaction.

### 2.1 Deep Learning Pipeline for E-Field Prediction

Recently, several deep-learning-based approaches have been developed for fast estimation [29,28,12]. While [29] did not consider subject-specific brain connectivity, the methods in [28,12] use subject-specific MRI scans and the magnetic field for whole-brain E-field prediction. [28] and [12] showed that using a deep neural network (*DNN*) can significantly reduce the prediction time of a whole-brain E-field from over 10 to 0*.*24 seconds. In this work, we follow the method in [28] by training a multi-scale 3D-ResUnet model with a reduced field-of-view to further accelerate the prediction time by 6 folds to achieve real-time E-field prediction. Details of our model training approach and data are available in (omitted, under review at MICCAI 2023).

### 2.2 Neuronavigation Software Component

**Fig. 2.**
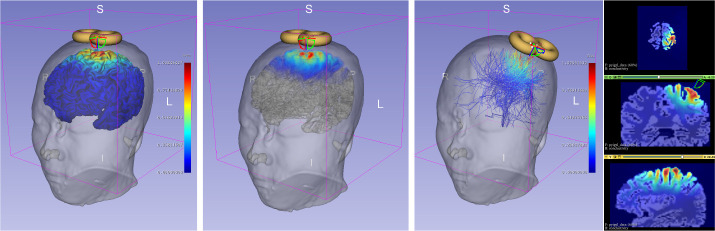
Brain Surface vs. Volume Rendering vs. Fiber Tractography in SlicerTMS. **Left**: E-field visualized in 3D on the gray matter surface of a subject with a figure-8 coil on top of the scalp. **Center**: E-field rendered on the volumetric data of the MRI scan. **Right**: SlicerTMS allows the user to add a full-brain dMRI or tractography with fiber tracts and then visualizes the electric field on the tractography data. SlicerTMS is showing the E-field on the according tracts. We added an interactively adjustable region of interest (ROI) for selecting specific fibers. Additionally, three 2D slices of the volume in axial, coronal, and sagittal directions show the E-field.

SlicerTMS provides real-time rendering of the predicted E-Field on a 3D brain surface mesh, its volume, and the brain’s white matter fiber bundles obtained from MRI scans to allow for patient-specific modeling. Our tool is currently integrated into the neuronavigation software 3D Slicer [7], utilizing Kitware’s VTK ^5^ framework to manipulate graphical elements within the rendered scene. SlicerTMS runs with any 3D Slicer version from 5.0.3 to the most current version. With SlicerTMS, users can visualize and explore real-time TMS results from standard desktop monitors on different operating systems. The data is transmitted from the DNN in real-time through a protocol called *OpenIGTLink*, a tool for network communication with external software or hardware using a protocol allowing real-time image and position streaming with submillisecond latency up to 1024 fps [25]. The DNN server component can run locally or as a remote service on a GPU server with data transfer via OpenIGTLink. Each time the neural network generates a new E-Field, it updates the TMS module. Inside SlicerTMS’ user interface, the user can manually adjust the coil by dragging and rotating it with the cursor into the desired position or using the text input fields to enter a coil position matrix where the 3D coil automatically moves to this position. While moving the coil, we inform the neural network via OpenIGTLink, generating the E-Field for this specific coil position. This process is shown in Figure 1. We created the coil as a 3D mesh in .stl format, simulating a ‘figure 8’ coil like the Magstim-70mm-Figure8 [24]. Users can freely use any other type of TMS coil by exchanging the example coil we provided with their coil file in the designated data folder. Equally, all other files, such as the skin, gray matter, and conductivity files, can be exchanged individually. 2D and 3D views of the neuronavigation component are illustrated in Figure 2.

### 2.3 Design of the AR Component with WebXR

SlicerTMS allows users to optionally start a web server that can either be redirected to the local browser or connect to a mobile device. Then, a secure WebSocket connection with the asynchronous networking library Tornado^6^ accesses objects from the neuronavigation platform 3D Slicer, transfers these to the browser, and vice versa. A modern smartphone with a depth sensor can control the TMS coil inside the 3DSlicer in real-time by rotating or moving the mobile phone (see video in Supplemental Material). Each time the phone moves, the position matrix updates via WebSocket to SlicerTMS. We use JavaScript to access the WebXR API to create an immersive web-based experience supporting both AR and VR [8]. Even if our primary goal was to implement mobile-based AR, WebXR allows VR headsets to access our real-time visualization as in Figure 1. The browser application uses the JavaScript framework ThreeJS^7^ for client-side scene rendering.

## 3 Evaluation and Validation

### 3.1 Dataset

For our experiments, we randomly selected ten different subjects from the Human Connectome Project (*HCP*) dataset [26] consisting of T1_*w*_ MRIs. The T1_*w*_ MPRAGE image was acquired with 0.7mm isotropic voxels. We replaced the conductivity file, skin, and brain mesh for each subject while the coil and deep learning model remained the same throughout all subjects.

### 3.2 Performance Experiments of SlicerTMS

To investigate the feasibility of SlicerTMS, we conducted different experiments to evaluate its performance given different scenarios. First, we tested our software using four different devices to guarantee the functioning of a complete real-time visualization. Then, we compared our tool to the current state-of-the-art alternative for TMS visualization in Section 3.3. We evaluated SlicerTMS based on both its performance of the E-field prediction and the real-time visualization. The results can be seen in Table 1. The input data consisted of an electric field of shape 70×90×50×1 generated by our trained model. The visualization consists of resampling the Nifti images and simultaneously projecting the E-field on a 3D brain mesh, brain volume, and the tractography data. We registered the time how long the code needs to execute each *run*, where a run is defined as the coil movement triggering the the neural network resulting in predicting an E-field that is immediately visualized in SlicerTMS. To get a precise measurement, we averaged the time of 50 runs. We had the following devices available: an Apple M1 MacBookBook Air with 16GB Memory (Apple M1), a workstation computer with an Intel Core i9–9980XE with 36 CPUs @ 3.00GHz and 64 GB RAM (CPU i9), an NVIDIA GeForce RTX 2080 GPU (2080Ti), and a remote NVIDIA A100 GPU (A100).

The results of our performance testing indicate that the neural network runs on average in less than 0*.*2 seconds and the real-time visualization in less than 10 milliseconds. We also measure performance on Apple’s arm-based M1 and M2 chips. However, Pytorch currently does not support 3D convolutions using Apple Metal Performance Shaders. Once available, we estimate a 3.5–15 times acceleration compared to their CPU without access to a (remote) GPU allowing portable TMS simulation.

### 3.3 Comparison to SimNIBS

**Fig. 3.**
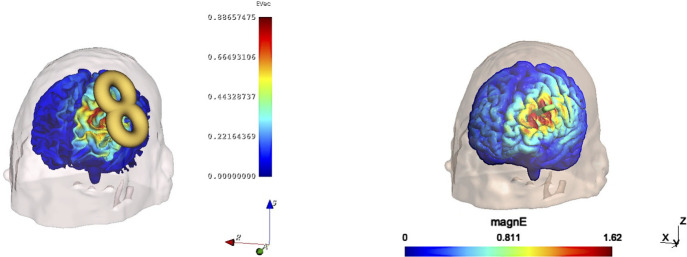
SlicerTMS vs. SimNIBS E-Field Visualization. **Left:** SlicerTMS shows E-field with movable Figure 8 coil, the color legend indicate strength of the E-field and axes with directions. **Right:** SimNIBS showing E-field on brain surface with coil direction (green handle).

We compared SlicerTMS to the existing and popular TMS visualization software SimNIBS [18,23]. However, unlike SlicerTMS, SimNIBS does not rely on deep learning but on visualizing an E-field based on manual coil placement. To compare both visualizations, we added the option in SlicerTMS to place a coil manually. We tested both tools on ten different subjects from the HCP dataset. We used SimNIBS v4.0 to compute E-fields induced by a Magstim-70mm-Figure8 coil at various locations and orientations. We programmatically measured the time only for the visualization part inside SimNIBS, then created the E-field for each of the ten subjects in random positions and entered these exact coil position matrices in SlicerTMS to generate the same E-field. Figure 3 shows how the same coil positions would be depicted in both tools. We did not measure the additional times SimNIBS requires, i.e., calculating the dA/dt field, computing matrices, or solving the system.

Table 2 shows the results of visualization speed comparison on two different CPU machines. SlicerTMS took, on average, 0*.*08506 seconds, while SimNIBS needs 7*.*58798 seconds to visualize an E-field on the brain. We conducted a two-sided t-test to determine if there was a significant difference in group means. The null hypothesis states no significant difference between SlicerTMS and SimNIBS (**H**_0_), while the alternative hypothesis states a significant difference between groups (**H**_1_). We used an independent samples t-test as the two groups are independent. The t-statistic was calculated to be *t*_38_ = 56.3, *p* < 0.0001, indicating a significant difference between the groups. We, therefore, reject **H**_0_, and accept the alternative hypothesis that there is a significant difference between SlicerTMS and SimNIBS.

## 4 Discussion and Future Work

The results from the experiments show that SlicerTMS is suitable for real-time visualization of TMS. Our experiments show that with a powerful GPU, we can run our complete application with a speed of around 0.16 seconds, including visualization and deep learning. This allows an almost smoothless frame rate. Furthermore, our experiments have shown that compared to the current state-of-the-art TMS visualization, SlicerTMS achieves a 78.83× faster performance speed. We were able to show the statistical significance with a t-test. Users must also be aware of network stability and latency when using a remote GPU or cloud solution. Still, our experiments on the remote A100 GPU show that running the deep learning network with remote inference can be faster than running locally (remote 0*.*15784s vs. local 0*.*26498s). This allows running SlicerTMS in a TMS clinic with remote hardware.

### Limitations and Caveats.

Despite our comparison, we want to emphasize that SimNIBS is a valuable tool for TMS visualization with additional functionalities not available in SlicerTMS. Our experiments focused on the real-time visualization aspects only. In the future, we plan to add more features to SlicerTMS, such as measuring the distance of the coil to the cortex, showing the vector fields, and improving the current WebXR face filter projecting the brain on a subject in AR.

## 5 Conclusion

This paper presents SlicerTMS, the first system for real-time visualization for TMS driven by a neural network for electromagnetic field prediction. We interface with the popular neuronavigation software 3D Slicer and add an immersive and web-based AR visualization component. Our visualization supports finding the optimal coil position to maximize targeted stimulation based on robuste prediction. SlicerTMS allows a local or remote GPU to run the deep learning model in real-time with a coupled visualization that is faster than existing software.

## Figures and Tables

**Table 1.** Performance Evaluation.

		Apple M1 mean±std. dev	CPU i9 mean±std. dev	2080Ti mean±std. dev	A100 (Remote) mean±std. dev	MEAN [s] mean±std. dev

**Subject 1**	**CNN**	3.03894 ± 0.11715	0.16642 ± 0.16238	**0.04028** ± 0.00565	0.05592 ± 0.01595	0.82539 ± 0.00773
	**Vis.**	**0.04755** ± 0.0019	0.09336 ± 0.01284	0.09555 ± 0.00825	0.09428 ± 0.01050	0.08254 ± 0.07528

**Subject 2**	**CNN**	3.04025 ± 0.07488	0.17434 ± 0.01121	**0.06735** ± 0.18290	0.06934 ± 0.01456	0.83398 ± 0.07089
	**Vis.**	**0.04682** ± 0.00084	0.09355 ± 0.01257	0.09865 ± 0.00683	0.09886 ± 0.01168	0.08448 ± 0.00798

**Subject 3**	**CNN**	3.03730 ± 0.0521	0.16703 ± 0.00911	**0.06516** ± 0.17955	0.06935 ± 0.09588	0.83471 ± 0.08416
	**Vis.**	0.04640 ± 0.00283	0.0949 ± 0.01431	0.09591 ± 0.01053	0.09699 ± 0.01376	0.08355 ± 0.01036

**Subject 4**	**CNN**	3.00502 ± 0.04971	0.16979 ± 0.00839	0.06585 ± 0.17467	**0.05568** ± 0.01793	0.82409 ± 0.06268
	**Vis.**	**0.05050** ± 0.01596	0.09094 ± 0.00856	0.09528 ± 0.01075	0.09636 ± 0.01216	0.08327 ± 0.01186

**Subject 5**	**CNN**	3.04525 ± 0.06020	0.17424 ± 0.00842	**0.0674** ± 0.18280	0.06773 ± 0.09635	0.83866 ± 0.08694
	**Vis.**	**0.04997** ± 0.01436	0.09611 ± 0.01756	0.09684 ± 0.00724	0.09721 ± 0.01347	0.08503 ± 0.01316

**Subject 6**	**CNN**	3.02331 ± 0.06009	0.17044 ± 0.01210	0.06975 ± 0.19970	**0.06397** ± 0.08236	0.83187 ± 0.08856
	**Vis.**	**0.04699** ± 0.00096	0.09695 ± 0.02807	0.09727 ± 0.01152	0.09359 ± 0.01363	0.08370 ± 0.01355

**Subject 7**	**CNN**	3.00740 ± 0.05809	0.17397 ± 0.00812	**0.06340** ± 0.17313	0.06527 ± 0.07967	0.82752 ± 0.07975
	**Vis.**	**0.04950** ± 0.01188	0.09514 ± 0.01094	0.09644 ± 0.01840	0.09620 ± 0.01194	0.08432 ± 0.01329

**Subject 8**	**CNN**	3.03198 ± 0.04614	0.16830 ± 0.01014	0.06624 ± 0.17561	**0.05417** ± 0.01574	0.83017 ± 0.06191
	**Vis.**	**0.04752** ± 0.00297	0.09478 ± 0.01425	0.09733 ± 0.00740	0.09691 ± 0.01182	0.08413 ± 0.00911

**Subject 9**	**CNN**	3.0257 ± 0.05572	0.17037 ± 0.00988	0.0657 ± 0.17642	**0.06445** ± 0.07943	0.83156 ± 0.08037
	**Vis.**	**0.04698** ± 0.0008	0.09527 ± 0.01315	0.09443 ± 0.00967	0.09537 ± 0.01228	0.08302 ± 0.00899

**Subject 10**	**CNN**	3.0236 ± 0.05616	0.16972 ± 0.01004	**0.06485** ± 0.17405	0.06586 ± 0.07994	0.83101 ± 0.08005
	**Vis.**	**0.05375** ± 0.02470	0.09406 ± 0.01384	0.09503 ± 0.01074	0.09631 ± 0.01201	0.08479 ± 0.01534

**MEAN [s]**	**(CNN+Vis.)**	3.08655 ± 0.07076	0.26498 ± 0.03959	0.15987 ± 0.01553	**0.15784** ± 0.07011	0.91479 ± 0.08826

We test SlicerTMS on ten different subjects and four different devices. Timings for E-field prediction (CNN) and visualization (Vis.) are shown separately for ten different subjects using four different hardware configurations. The remote A100 setup using cloud-based inference is the fastest. All times in seconds.

**Table 2.** Comparison with SimNIBS.

	SimNIBS			SlicerTMS			
	Apple M1 [s]	CPU i9 [s]	Mean [s]	Apple M1 [s]	CPU i9 [s]	Mean [s]	Improvement

**Subject 1**	6.81622	6.43095	6.62369	0.05539	0.09607	0.09607	66.67x faster
**Subject 2**	8.22622	7.98845	8.10733	0.06353	0.09891	0.09891	81.97x faster
**Subject 3**	7.69764	7.37311	7.53538	0.05733	0.09138	0.09138	82.46x faster
**Subject 4**	7.08191	7.08276	7.08233	0.08974	0.10517	0.10517	67.34x faster
**Subject 5**	7.72296	6.60987	7.16642	0.12807	0.09652	0.09653	74.24x faster
**Subject 6**	7.91043	7.43637	7.67339	0.04735	0.09448	0.09448	81.21x faster
**Subject 7**	8.80702	8.10869	8.45786	0.06146	0.10163	0.10163	83.22x faster
**Subject 8**	7.90561	7.3814	7.64351	0.05229	0.09645	0.09645	79.25x faster
**Subject 9**	8.1296	8.0317	8.08065	0.12303	0.09769	0.09769	82.71x faster
**Subject 10**	7.2251	7.79360	7.50936	0.06049	0.084202	0.08420	89.18x faster

**Mean**	7.75227	7.42369		0.07387	0.09625		

**Mean ± std.dev.**	7.58798 ± 0.59553			0.08506 ± 0.02365			**78.83x faster**

We measure visualization speed of an E-field on the brain mesh at fixed TMS coil positions in both tools. We report measurements for two hardware configurations. All timings in seconds. SlicerTMS is over 78 × faster.

## Footnotes

5
https://vtk.org/

6
https://www.tornadoweb.org/

7
https://threejs.org/
